# YEARS WITH AND WITHOUT HOME-BASED SERVICES FOR THE 70+ POPULATION IN NORWAY: TRENDS OVER THREE DECADES, 1995–2019

**DOI:** 10.1093/geroni/igac059.101

**Published:** 2022-12-20

**Authors:** Milan Chang Gudjonsson, Mona Michelet, Bjørn Heine Strand

**Affiliations:** Norwegian Institute of Public Health, Reykjavik, Iceland; Vestfold Hospital Trust, Oslo, Oslo, Norway; Norwegian Institute of Public Health, Oslo, Oslo, Norway

## Abstract

**Introduction:**

Life expectancy (LE) is increasing worldwide, while there is a lack of information on years of home-based formal care use among the aging population. The current study examined the trend of LE for formal care use among Norwegian older adults over three decades 1995-2016 in Norway.

**Methods:**

A total of 25,263 participants aged 70+ were included in the Trøndelag Health Study (HUNT) survey 2 (1995-97), 3 (2006-08), and 4 (2017-19). Participants reported the use of formal care services including practical help and home nursing. The prevalence of both service uses was standardized to the Norwegian population by age, sex, and education using post-stratification weights. LE was estimated using National mortality data by age, sex, and education combined with the formal service use data using the Sullivan method to estimate expected years with and without basic services and nursing services in Norway.

**Results:**

During 1995/97-2017/19, the service use decreased from 22.6% to 6.2% for practical help and 6.4% to 5.5% for receiving home nursing. LE at age 70 from 1995 to 2016 increased 3.4 years in men, and 2.4 years in women. Expected years receiving practical help decreased by 1.1 years (2.6 to 1.5 years) in men and 1.6 years (4.5 to 2.9 years) in women, while LE for home nursing increased from 0.7 years (0.7 to 1.4 years) in men and 1.3 years (1.4 to 2.7 years) in women.

**Conclusions:**

Years receiving home nursing increased during 1995-2016, while years receiving practical help care decreased in the older Norwegian population.

